# Reduced methane-bearing fluids as a source for diamond

**DOI:** 10.1038/s41598-020-63518-2

**Published:** 2020-04-24

**Authors:** Vladimir Matjuschkin, Alan B. Woodland, Daniel J. Frost, Gregory M. Yaxley

**Affiliations:** 1 Institut für Geowissenschaften, Goethe-Universität Frankfurt am Main, Altenhöferallee 1, 60438 Frankfurt am Main, Germany; 2 Bayerisches Geoinstitut, University of Bayreuth, Universitätsstraße 30, 95447 Bayreuth, Germany; 3 Research School of Earth Sciences, The Australian National University, Canberra, ACT 2601 Australia

**Keywords:** Geochemistry, Mineralogy, Petrology

## Abstract

Diamond formation in the Earth has been extensively discussed in recent years on the basis of geochemical analysis of natural materials, high-pressure experimental studies, or theoretical aspects. Here, we demonstrate experimentally for the first time, the spontaneous crystallization of diamond from CH_4_-rich fluids at pressure, temperature and redox conditions approximating those of the deeper parts of the cratonic lithospheric mantle (5–7 GPa) without using diamond seed crystals or carbides. In these experiments the fluid phase is nearly pure methane, even though the oxygen fugacity was significantly above metal saturation. We propose several previously unidentified mechanisms that may promote diamond formation under such conditions and which may also have implications for the origin of sublithospheric diamonds. These include the hydroxylation of silicate minerals like olivine and pyroxene, H_2_ incorporation into these phases and the “etching” of graphite by H_2_ and CH_4_ and reprecipitation as diamond. This study also serves as a demonstration of our new high-pressure experimental technique for obtaining reduced fluids, which is not only relevant for diamond synthesis, but also for investigating the metasomatic origins of diamond in the upper mantle, which has further implications for the deep carbon cycle.

## Introduction

Diamond formation in the Earth has been extensively discussed in recent years based upon analysis of natural materials, high-pressure experimental studies, or theoretical considerations. Some researchers consider diamond to form by direct transformation from graphite with increasing pressure (i.e. through subduction)^1^. This is also the basis of numerous industrial studies^2^. Experimental investigations indicate that a large overstep in pressure and/or temperature above the univariant graphite-diamond phase boundary is generally required to efficiently drive the reaction (i.e. 12–25 GPa and 1600–2500 °C^3^) compared to equilibrium conditions near 5 GPa at 1100–1500 °C that were determined by observing diamond growth on seed crystals^2,4^. The later conditions are also consistent with those recorded by coexisting pairs of mineral inclusions in some natural diamonds^5,6^. However, diamond crystallisation from graphite at 5–7 GPa has proved unsuccessful without the presence of seed crystals^7^. Evidence for mantle-derived diamonds having formed by direct transformation from graphite is rare, although graphite does occur as an inclusion in natural diamond^8^.

On the other hand, many studies support a metasomatic origin, crystallizing from C-bearing fluids or melts migrating through the lithospheric mantle^6,8,9^. The character of the metasomatic agent is very controversial and it is likely that a number of different reactions leading to diamond formation occur locally. Metasomatic crystallization of diamond from C-bearing fluids or melts is generally considered the most common mechanism for diamond formation^8,9^ and two essentially mutually exclusive mechanisms can be considered, both of which involve redox reactions: i) the reduction of oxidized carbon in the form of CO_2_ or carbonate, or ii) the oxidation of reduced carbon from methane or other higher hydrocarbons. In fact, the ^13^∂C and N systematics of natural diamonds suggest that both mechanisms might be responsible for the formation of diamonds in different mantle lithologies^8–11^.

Direct reduction of CO_2_-rich fluid to form diamond in either a peridotitic or eclogitic mineral assemblageis is a possible mechanism, however, appears to be unlikely under the pressure-temperature and redox conditions of the deep cratonic lithospheric mantle (P > 5 GPa) due to the relative stability of carbonate minerals compared to CO_2_-rich fluids, as emphasized by Luth^12^. Diamond formation by reduction of oxidized C could involve either carbonate minerals or a carbonate-bearing melts^13,14^. The oxidation state of the mantle at depths where diamonds form (i.e. >~150 km) generally lies below the minimum oxygen fugacity (ƒO_2_) for carbonate stability^15^ as defined by equilibria such as enstatite + magnesite = forsterite + C (diamond) + O_2_ (EMOD)^16^. Therefore, carbonate minerals or melts may exist locally at pressures above 5 GPa, either where oxidizing metasomatism has occurred, or potentially within subducting slabs that are isolated from the ambient mantle. On the other hand, the rare occurrence of carbonate inclusions in diamond provides direct evidence for some diamonds having formed through carbonate reduction^17,18^. Under ƒO_2_ conditions where pure carbonatite melt would be unstable, metasomatic melts may have a mixed carbonate-silicate character^19^. Spontaneous diamond crystallization through reduction of such a melt was recently demonstrated experimentally by Girnis *et al*.^20^ in a model peridotite-sediment system. Whether or not such processes are generally responsible for diamond formation in the Earth’s mantle remains open to debate. Our contribution here addresses an alternative mechanism that is likely to be important for the Earth, as described below.

The formation of diamond from reduced methane-rich fluids is a further possibility that has a number of merits. For example, the ambient ƒO_2_ of the lithospheric mantle at depths where diamond becomes stable (i.e. ~150 km) lies well below the stability of CO_2_-rich fluids or carbonatitic melts^15^. In addition, some diamonds exhibiting negatively skewed ^13^∂C signatures^8,10,21^ contain CH_4_ ± H_2_-bearing fluid inclusions, as detected by Raman spectroscopy^22,23^). These studies provide direct evidence for the role of CH_4_ in the formation of some natural diamonds, including the population of very large “CLIPPIR” diamonds^24^, even so diamond synthesis from strongly reduced fluids has not yet been experimentally observed^25^. There are further reasons to suspect that the mechanism of diamond crystallization through CH_4_ oxidation may be more prevalent than previously recognized. Aside from CH_4_ having more than double the carbon carrying capacity of carbonates or CO_2_ (75 wt.% C in CH_4_ versus 27 wt.% in CO_2_ and 12 wt.% in CaCO_3_), the solubility of CH_4_ in silicate melts is very low, on the order of 100–500 ppm even under conditions of unit activity of CH_4_^26^. Thus, the depression of the peridotite solidus temperature is much less than in the presence of more oxidized H_2_O-CO_2_-rich fluids^27^. As a result, CH_4_ (±C_2_H_6_, ±H_2_) might be the only viable “free” fluid phase stable in the deeper parts of the upper mantle over a large range of temperature and depth. However, some thermodynamic models suggest that the stability of CH_4_-rich fluids requires redox conditions so reducing that the ƒO_2_ must lie below that of metal saturation (i.e. below the Ni-precipitation curve, which lies just below the iron-wüstite (IW) oxygen buffer^15,28^). This could call into question the relevance of such reduced fluids for the formation of diamond in the upper mantle since the Ni-precipitation curve effectively places a lower limit on the feasible ƒO_2_, even if rare moissanite inclusions have been reported^29^. Furthermore, CH_4_ may be unstable in the presence of metals as they may react to form carbides (e.g. FeSiC alloy or (Fe, Ni)_3_C)^30^.

To investigate the potential conditions under which diamond can form from methane-rich fluids, we have undertaken a series of experiments at pressures and temperatures corresponding to the deeper portions of the cratonic mantle lithosphere under controlled ƒO_2_. A pressure range of 5–7 GPa is of particular interest as this is similar to the range reported for many lithospheric diamonds^21^ and where no solid-phase transformation of graphite to diamond is expected (graphite has long been used as a heater for experiments at these pressures without spontaneous transformation). No diamond seed crystals were used to initiate or accelerate diamond growth^2^. Although previous experimental studies have had little to no success in forming diamond at such conditions without diamond seeds^25,31,32^ our experiments followed the approach of Matjuschkin *et al*.^33^, comprising a harzburgitic mineral assemblage of natural olivine and orthopyroxene packed into an olivine capsule along with a coexisting COH-fluid (see Methods). Possible reasons why diamond synthesis in the presence of methane was unsuccessful are briefly discussed in the supplementary information. The ƒO_2 _imposed on the sample was measured post-experiment using an Ir-Fe redox sensor^34^. Spectroscopic analysis of the run products provides important insights into the nature of the resulting fluid and mineral phases, including the unequivocal identification of spontaneous diamond formation in our experiments.

## Results and discussion

**Figure 1:**
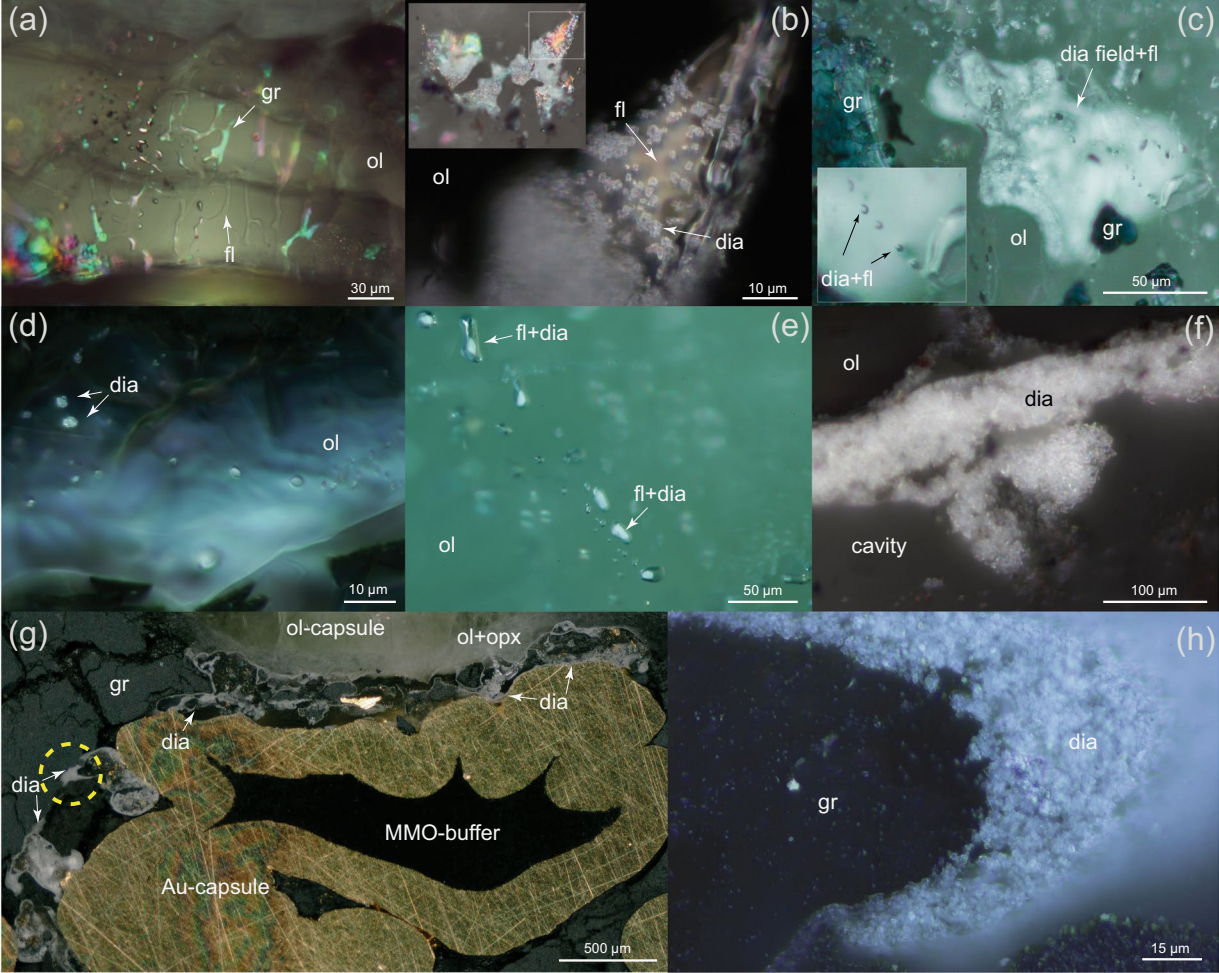
Examples of run products. In (**a**) a graphite-bearing (gr) experiment at 5 GPa, 1280 °C with methane-rich fluid (fl) channels in olivine (ol). (**b**) Diamond-bearing fluid inclusion in an experiment run at 7 GPa and 1250 °C. Diamonds (dia) occur as 1–4 µm single grains, or as aggregates. Note that graphite is not present inside the inclusion, but next to it. (**c–e**) are from a single experiment at 7 GPa, 1300 °C. (**c**) A ~100 µm large diamond pocket and fluid inclusions containing ~1–2 µm diamonds. (**d**) Diamond inclusions in olivine without associated fluid. (**e**) Diamonds up to 8 µm across coexisting with fluid. Similar to that depicted in (**b**), no graphite is present in these fluid inclusions, suggesting that diamond forms by precipitation from the fluid and not via a solid-solid phase transformation. (**f**) Diamond pocket along a crack in olivine produced at 5 GPa and 1250 °C. (**g**) formation of diamond vein in graphite around the buffer capsule. (**h**) A fragment of a diamond-rich zone highlighted in (**g**) at high magnification illustrates the formation of rounded diamond crusts with variable grain size. Corresponding Raman spectra for fluids and diamond in (**b**), (**f–h**) are presented in the supplementary materials.

**Figure 2:**
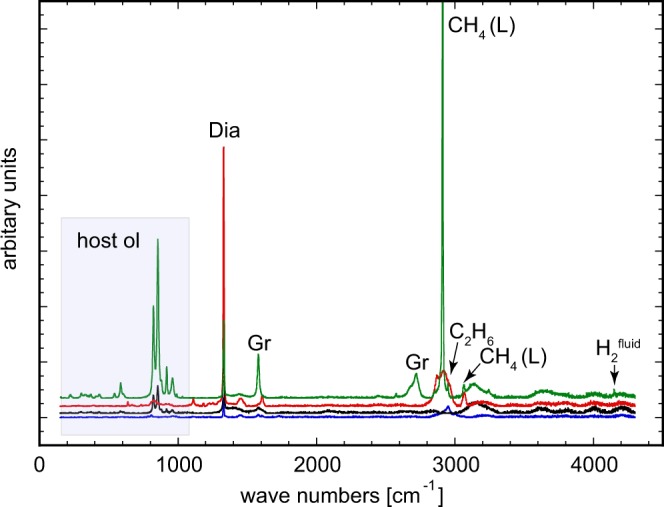
Representative Raman spectra of several diamond-bearing fluid inclusionsin olivine. The uppermost spectrum (green) was obtained in non-confocal mode to sample a larger volume of olivine (hence the stronger signal from olivine). In this way, we were able to detect H_2_ in the fluid. This also meant that both graphite and diamond were detected, although they were located at different depths within the olivine and not in direct contact with each other (green spectrum only).

**Figure 3:**
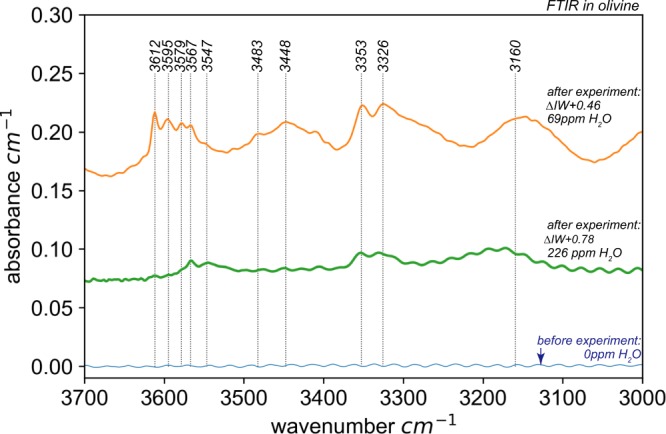
Unpolarised FTIR spectra of the initially anhydrous San Carlos olivine capsule material compared with those take after experiment 1585 and 1583^33^. Within the range of 3000–3700 cm^−1^, where absorption due to OH stretching is expected^41,56^, the olivine capsule material exhibited no measurable intensity (i.e. essentially no initial OH). The water concentrations are representative for the entire olivine crystal and reveal incorporation of OH in olivine via essentially all four different substitution mechanismsas reported by^41^. The thickness of 1585 and 1583 thin section are 85 and 190 µm respectively. Note that the OH contents of this study are lower compared to those observed by Sokol *et al*.^57^, in similar experiments, which we ascribe to their experiments having higher ƒO_2_ (and ƒH_2_O) compared to conditions of our runs.

The Ir-Fe redox sensors gave values of 0.2–0.8 log units above the Fe-FeO (IW) oxygen buffer (i.e. ∆logƒO_2_ = IW + 0.2 to IW + 0.8, see supp. info Table S2), indicating that our experiments were carried out above FeNi-alloy saturation and at similar to ∆logƒO_2_ values reported for some mantle xenoliths originating from ≥150 km depth^19,35,36^. In our experiments, the coexisting fluid phase was effectively trapped in a network of inclusions within the olivine capsule at pressure and temperature (Fig. 1a,b), permitting its composition to be directly probed by Raman spectroscopy (see Methods). While quantitative assessment of the fluid composition was not feasible, spectra reveal fluids composed essentially of CH_4_ with minor C_2_H_6_ and H_2_ (Fig. 2, see also Matjuschkin *et al*.^33^ and Fig. S1 in supp. info.). Although a number of commonly used thermodynamic models for COH-fluids^37,38^, including GFluids^28^, predict a significant H_2_O component (up to 40 mol %) at the P-T-ƒO_2_ conditions of our experiments, virtually no H_2_O was detected in the Raman spectra in spite of an extensive search across the samples. The absence of different inclusion populations means that there is no evidence for liquid immiscibility between CH_4_ and H_2_O. Our observations imply that CH_4_ is much more stable than most models predict and is likely to be a major component of COH fluids at significantly higher ƒO_2_ values than generally thought. On the other hand, our results are consistent with the fluid speciation model of Huizenga^39^ that predicts ~5 mol % H_2_O at the conditions of our experiments (see supp. info Fig. S1), as such low concentrations might not be detectable in Raman spectra^40^. A finite amount of H_2_O in the fluid phase is not only expected on theoretical grounds (i.e. there must be a finite thermodynamic activity of H_2_O) but is required by the presence of OH as detected in olivine by FTIR spectroscopy (Fig. 3). In our experiments, the amount of OH in olivine increases with increasing ƒO_2_ and is related to a concomitant increase in water activity^41^ (Fig. 3). While the quantitative assessment of OH concentrations in olivine is beyond the scope of this contribution, the observed incorporation of OH into olivine has important implications for the mechanism of diamond formation as well as the composition of the coexisting fluid in our experiments (see below).

Along with the CH_4_-rich fluid inclusions in olivine, diamond was also observed in many experiments and confirmed by Raman spectroscopy (Figs. 1a–c, 2). The diamonds exhibit a Raman line at 1332 cm^−1^, which is the ideal value for well crystallized natural diamond^42^. As no diamond seeds were employed, their presence must be the result of spontaneous nucleation during the experiments. The possibility that the diamond could have been introduced during sample preparation can be ruled out since: i) all diamond-bearing samples were polished with an Al_2_O_3_ slurry rather than with diamond paste, and ii) many diamonds including those illustrated in Fig. 1b–e occur well beneath the sample surface. The diamonds are 1–5 µm in size and occur in a variety of textures: type 1) as single-crystals or as polycrystalline inclusions in olivine (Fig. 1d), type 2) within fluid inclusionsin the absence of graphite (Fig. 1b,c,e), type 3) in diamond-rich zones or veins at the interface between olivine-orthopyroxene sample material and the outer Au-capsule (Fig. 1f) or type 4) as concentrations at or near the contact with the inner buffer capsule that supplies H_2_ to the sample (Fig. 1g,h). These different types of occurrence emphasize the mobility of CH_4_-fluids, driven in part by unavoidable, but small axial thermal gradients, probably of the order of a few degrees across the capsule.

Diamond was observed in experiments performed at 5, 6 and 7 GPa (Fig. S2 supp. info.). At both 7 and 6 GPa and temperatures from 1050 to 1300 °C diamond crystallized in all experiments, even one that had only a 4 hours duration. The diamond yield appears to increase with the experiment duration, although, it is difficult to quantify this since only a small amount of fluid was initially added (4 wt%, see Methods) and the spatial distribution of diamond is uneven. It is in fact quite remarkable that such a small amount of fluid is capable of producing spontaneous diamond precipitation. At 5 GPa only one experiment run at 1250 °C was found to contain diamond (see Supplementary Table S1). The diamond yield was less than observed in experiments at 6 and 7 GPa, which we ascribe to the very close proximity to the graphite-diamond phase boundary^2^.

Although graphite is also present, it formed at the onset of the experiment by the breakdown of the stearic acid, which served as the source of the COH fluid^43^. In most cases, diamond in fluid inclusions is not observed to have any direct textural association with graphite (texture types 1, 2, 3 described above). This means that diamond must have crystallized from the methane-rich fluid itself rather than by solid-state transformation of graphite with diamond precipitating from the CH_4_-fluid as it migrated along cracks in olivine or between the sample and the outer or inner capsule. Where diamond crystallized at or near the surface of the inner buffer capsule (textural type 4), the diamond aggregates developed upon the outer margins of graphite clots (Fig. 1g,h). This spatial relationship suggests an essential role of the fluid phase and the proximity to a source of H_2_ in diamond formation. This interpretation is consistent with the observations of Akaishi *et al*.^31^ who proposed a dissolution-reprecipitation mechanism for the crystallization of diamond in their graphite-fluid experiments based upon isotopic labelling of the carbon.

Diamond formation can be considered as an oxidation reaction either directly involving oxygen

1 $${{\rm{CH}}}_{4}+{{\rm{O}}}_{2}={{\rm{C}}}^{{\rm{dia}}}+2{{\rm{H}}}_{2}{\rm{O}}$$

or involving removal of hydrogen

2 $${{\rm{CH}}}_{4}\rightleftarrows {{\rm{C}}}^{{\rm{dia}}}+2{{\rm{H}}}_{2}$$

3 $${{\rm{C}}}_{2}{{\rm{H}}}_{6}\rightleftarrows {{\rm{CH}}}_{4}+{{\rm{C}}}^{{\rm{dia}}}+{{\rm{H}}}_{2}$$

The formation of diamond via equilibrium reaction 1 requires an oxygen source, which could be coupled with the reduction of Fe^3+^ to Fe^2+^. As discussed by Stachel and Luth^6^, the Fe_2_O_3 _content of upper mantle garnet peridotite is relatively small, limiting the supply of oxygen for such a process. In our experiments, some oxygen could be provided by the natural orthopyroxene in the starting materials that has Fe^3+^/∑Fe = 0.09(2), as determined by Mössbauer spectroscopy (see Methods). Considering the redox conditions of our experiments, reactions 2 and 3 should be more relevant where diamond forms upon removal of H_2_. One way for this to happen is for H to become sequestered in orthopyroxene and olivine via the equilibria proposed by Tollan and Herman^44^ for orthopyroxene:

4 $$3\,{{\rm{Fe}}}_{4/3}^{3+}[]{{\rm{Si}}}_{2}{{\rm{O}}}_{6}+0.5\,{{\rm{Fe}}}_{2}{{\rm{Si}}}_{2}{{\rm{O}}}_{6}+2\,{{\rm{H}}}_{2}+{{\rm{H}}}_{2}{\rm{O}}=3\,{{\rm{Fe}}}^{2+}{{\rm{H}}}_{2}{{\rm{Si}}}_{2}{{\rm{O}}}_{6}+{{\rm{Fe}}}_{2}{{\rm{SiO}}}_{4}$$

5 $$({{\rm{Fe}}}^{2+}{{\rm{Fe}}}^{3+})({{\rm{Fe}}}^{3+}{\rm{Si}}){{\rm{O}}}_{6}+0.5\,{{\rm{H}}}_{2}={({{\rm{Fe}}}^{2+})}_{2}({{\rm{Fe}}}^{3+}{\rm{H}}){{\rm{SiO}}}_{6}$$

and by Tollan *et al*.^45^ for olivine:

6 $$3\,{{\rm{Fe}}}_{4/3}^{3+}{{\rm{SiO}}}_{4}+{{\rm{H}}}_{2}+4\,{{\rm{H}}}_{2}{\rm{O}}=3\,{{\rm{Fe}}}^{2+}{{\rm{H}}}_{2}{{\rm{SiO}}}_{4}+{{\rm{FeH}}}_{4}{{\rm{O}}}_{4}$$

7 $${{\rm{Fe}}}^{2+}{{\rm{Fe}}}_{2}^{3+}{{\rm{O}}}_{4}+2\,{{\rm{H}}}_{2}+4\,{{\rm{H}}}_{2}{\rm{O}}=3\,{{\rm{Fe}}}^{2+}{{\rm{H}}}_{4}{{\rm{O}}}_{4}$$

The [] in equilibrium (4) denotes a lattice vacancy in orthopyroxene. The formation of OH in olivine during the experiments is documented by the FTIR spectra presented in Fig. 3. Unfortunately, the orthopyroxene grains were too small to analyse spectroscopically, but must also contain OH. Thus, the hydroxylation reactions (4–7) will act to drive reactions (2) and (3) to the right, promoting diamond formation. Such a mechanism should operate in the upper mantle as CH_4_-bearing fluids migrate into “drier” domains, such as those observed in the deeper portions of cratonic roots^46^. These mechanisms require the presence of contrasting mantle domains (i.e. dry vs. fluid-rich).

With ƒH_2_ internally buffered in our experiments, it might be expected that equilibrium 2 and 3 would shift to the left and destabilise diamond^15^. However, this is not supported by the occurrence of euhedral diamonds within CH_4_-rich fluid inclusions (Fig. 1b,c,e) and the crystallization of diamond near the interface with the inner buffer capsule where a H_2_ flux is expected (Fig. 1g,h). In fact, H_2 _and CH_4 _may play an essential role in a more complex process where metastable graphite is dissolved into the fluid in form of CH_4 _only to supersaturate and precipitate the more stable diamond. In this way, equilibria 2 and 3 shift to the left in contact with graphite and then shift to the right crystallizing diamond as ƒH_2_ is locally lowered. This mechanism is consistent with the observed preferential “etching” of graphite by H_2 _and CH_4 _compared to diamond^31,47^. The presence of H_2_ and CH_4_ is also known to stabilize the surface of diamond and promote sp^3 ^molecular orbital hybridization of carbon, thus promoting diamond growth^48^. Graphite etching is a well-known process in the physics literature^49,50^ and can explain the textural occurrence of our type 4 diamond and type 2 diamond-bearing fluid inclusions (Fig. 1b,c,e,g,h). We note that this process can generate diamond at essentially constant temperature, pressure and ƒO_2_, even at sub-solidus conditions. The relevance of such a process in nature can be found in the interaction of subducted graphite^51,52^ with CH_4_ and H_2_-bearing reduced fluids that may be generated by high-pressure metamorphism of ophiocarbonates (carbonate-bearing ultramafic rocks) in the subducting slab^52,53^. Subducted components and lithologies have frequently been implicated in diamond formation^1,54^. Determining if natural diamond crystallised from reduced fluids is unfortunately problematic in the absence of coexisting fluid inclusions. Even if such inclusions are present, their composition is most likely to have been modified during transport. Significant loss of H_2_ and CH_4_ from olivine can also occur during sample preparation (heating and vacuum conditions), unlike OH defects in olivine that can be observed by FTIR measurements.

In addition to the afore-mentioned mechanisms, we observe two further processes that are relevant for the mantle environment. In addition to the presence of OH groups (Fig. S2), Matjuschkin *et al*.^33^ report Raman spectra that also indicate incorporation of H_2_ into olivine rather than just in fluid inclusions, as was first described by Yang & Keppler^55^. This provides a further mechanism to crystallize diamond by driving both equilibria (2) and (3) to the right. Yang and Keppler^55^ report a minimum of 15–40 ppm molecular H_2_ residing on interstitial sites of olivine and orthopyroxene (and clinopyroxene) at 2.5 GPa and suggest that 100’s of ppm could be incorporated at higher pressures.

Secondly, significant cooling (e.g. from 1200 to 850 °C) will also promote diamond precipitationas the speciation changes and more H_2_O is formed (Fig. S1). This is not only the case for more oxidizing conditions near the “water maximum” as described by Stachel and Luth^6^, but also for CH_4_-rich fluids, as predicted by the speciation model of Huizenga^39^ and documented experimentally by Matjuschkin *et al*.^33^. The amount of diamond precipitation is not only a function of the incremental temperature decrease, but is also related to the final temperature and ƒO_2_ of the fluid (see Supplementary Data, Fig. S1). Depending on the C-species in the fluid, this process is essentially redox neutral.

Since ƒH_2_ and thus ƒO_2_ were held constant in our experiments by an internal buffer (see Methods), and pressure and temperature were also kept constant, the observed spontaneous formation of diamond is not related to any significant shift in redox state. Instead, diamond crystallization occurs from very CH_4_-rich fluids by a variety of processes, involving interactions between H_2 _and olivine, pyroxene or graphite “etching” in contact with H_2_ and CH_4_. Such fluids are stable at pressures and temperatures similar to those expected in the upper mantle at ≥150 km and at realistic ƒO_2_ conditions above metal saturation^33^. In addition, their rather weak effect on depressing the peridotite solidus^26^ means that CH_4_-rich fluids are likely to exist along a range of geothermal gradients in the deeper lithospheric mantle and in sublithospheric domains without being quantitatively extracted into a melt phase. This is consistent with the detection of CH_4_ and H_2_ associated with inclusions in the large sublithospheric “CLIPPIR” suite of diamonds^24^. The implication is that CH_4_-rich fluids are not only more prevalent in nature than often thought, but that they may represent a significant source of carbon responsible for diamond formation and that the associated H_2_ plays an important role in this process. Thus, this study not only confirms the potential importance of methane in the formation of diamond via several unanticipated mechanisms, but also suggests a high probability for diamond formation at mantle conditions through the involvement of methane-rich fluids. That implies that low density methane-rich fluids play a larger role in a deep carbon cycle as previously appreciated.

## Supplementary information

Supplementary information.
