# Supplementary material for: Reduced methane-bearing fluids as a source for diamond
Source: Sci Rep. 2020 Apr 24;10:6961. doi: 10.1038/s41598-020-63518-2 (PMC7181848; doi:10.1038/s41598-020-63518-2)
Supplement: Supplementary file 1 — Supplementary information. [file 41598_2020_63518_MOESM1_ESM.pdf]

Reduced methane-bearing fluids as a source for diamond

Vladimir Matjuschkin[1], Alan B. Woodland[1], Daniel J. Frost[2], Gregory M. Yaxley[3]
[1] Institut für Geowissenschaften, Goethe-Universität Frankfurt am Main, Altenhöferallee 1, 60438 Frankfurt am Main, Germany
[2] Bayerisches Geoinstitut, University of Bayreuth, Universitätsstraße 30, 95447 Bayreuth, Germany
[3] Research School of Earth Sciences, The Australian National University, Canberra, ACT 2601, Australia

Corresponding Author: Vladimir Matjuschkin, email: vladimir.matjuschkin@gmail.com

Supplementary information

Experimental procedures. High-pressure experiments were carried out using a belt-apparatus at the Goethe-Universität Frankfurt and a large volume multi-anvil press at the Universität Bayreuth. The experimental assembly follows the design of Matjuschkin et al. [33] and consists of (i) an outer Au capsule containing the mineral starting material (Table S1), (ii) a reduced fluid source (e.g. stearic acid), (iii) an inner capsule containing a reduced metal buffer assemblage (Fe-FeO or Mo-MoO_2) plus H_2O, as well as (iv) an outer pressure cell made of a material (CaF_2) to provide a hydrostatic environment and to minimize H_2-loss from the capsule. The fO_2 inside the outer capsule was determined using the Ir-Fe redox sensor technique [34] as is reported in Table S2. Part of the starting material was packed into a single-crystal capsule of San Carlos Olivine that was initially anhydrous (~0 ppm H_2O, see Figure S2). This capsule was used to trap fluid inclusions that could be investigated ex situ by Raman spectroscopy. Details are provided in a separate manuscript by Matjuschkin et al. [33]. The buffer assemblage was always detected by microprobe investigation after the experiment. An example of such an image is provided in Matjuschkin et al. [33].

Amount of fluid added was calculated from the decomposition reaction of stearic acid: $C_{18}H_{36}O_2 = 8\ CH_4 + 2\ H_2O + 10\ C$ [32, 43]. (In practice, a small amount of C_6H_6 may also form [31]). The amount of CH_4 and H_2O is 58 wt% of the total mass of stearic acid added, or ~4wt% of the total sample mass (i.e. ol+opx, buffer excluded). The graphite-saturated CH_4-H_2O bearing fluid produced at the onset of each experiment was reduced to ~IW+0.2 to IW+0.8 (Table S2). The previous study of Matjuschkin et al. [33] demonstrated that these fO_2 conditions could be maintained for at least 24 hours and that CH_4 remains in equilibrium with a harzburgite (±cpx, ±grt) and silicate melt (1-3%) during this time. Following the model of Huizenga [39] and elaborated by Matjuschkin et al. [33], the fluid composition should exhibit only a limited variation over the

temperature range of our experiments (Figure S1). A significant change in CH_4 mole fraction only occurs upon cooling below 1000°C and the extent is fO_2-dependent.

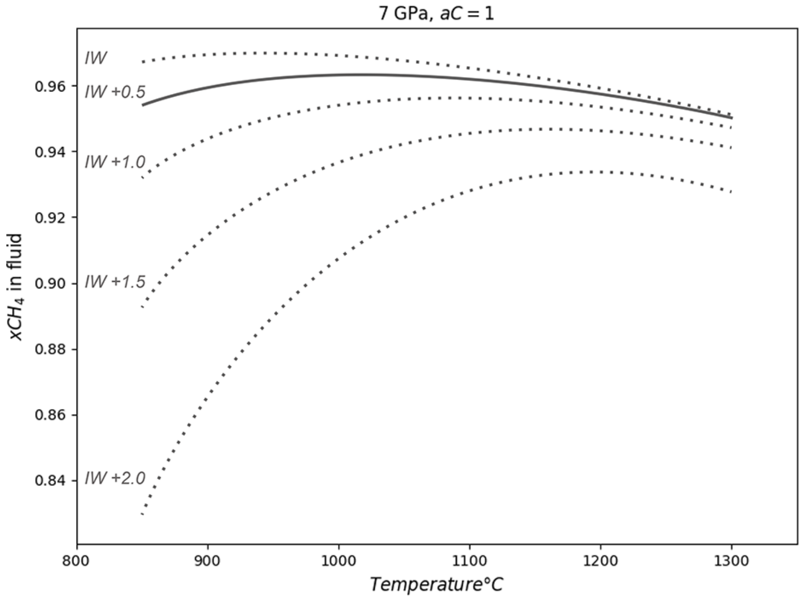

Figure S1: Fluid composition calculated from the model of Huizenga [39] at 7 GPa, for $\Delta \log fO_2$ between IW and IW+2 for unit activity of carbon (a_C=1). Note that decreasing temperature only has a significant effect on fluid composition at $\Delta \log fO_2$ > IW+1.0 and T <1100°C. At IW+0.5 (solid line) the fluid has a methane mole fraction of 0.96, similar to experimental results reported in this study and by Matjuschkin et al. [33].

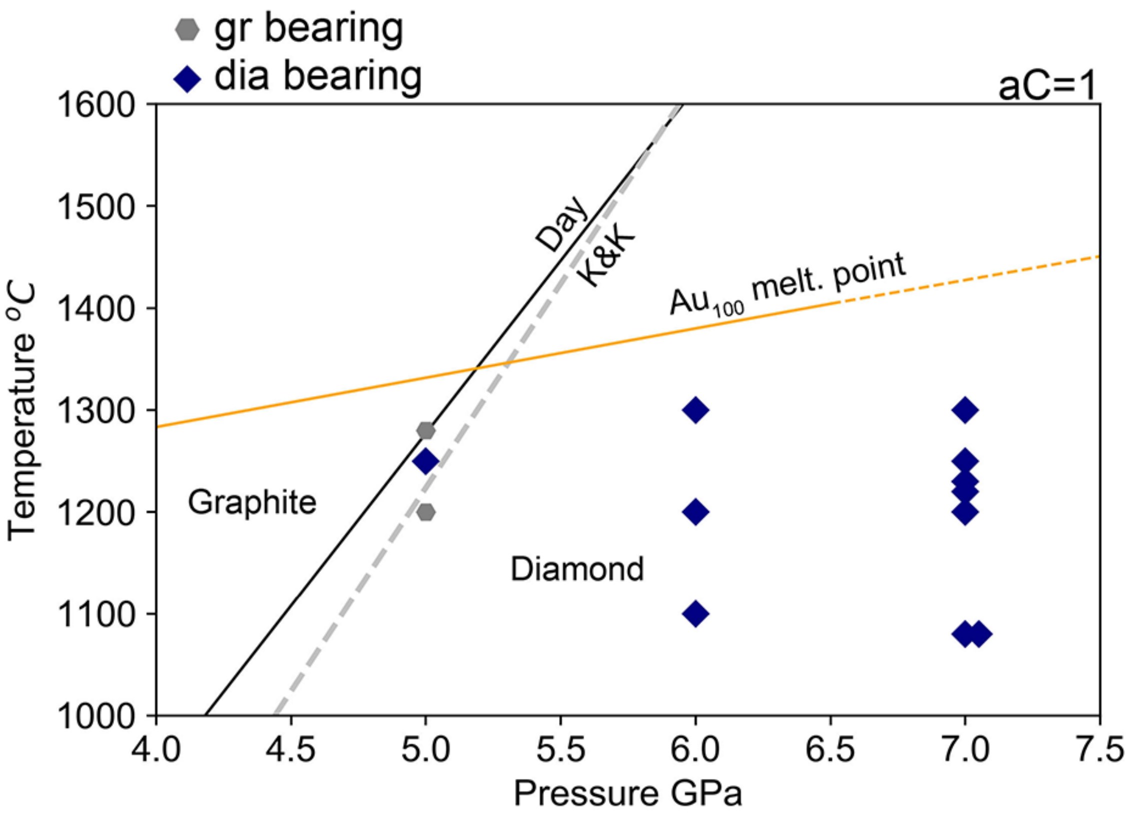

Figure S2: Diamond-bearing experiments in a P-T-field. The graphite-diamond boundary was calculated after Kennedy&Kennedy [2] and Day [4] and melting point of pure gold after by Akella [58].

Analytical techniques. The recovered capsules were mounted in epoxy and ground down to expose the sample material. They were then polished with paraffin, Al_2O_3 since liquid oils can penetrate deep into graphite pockets and is difficult to remove, even when placed under vacuum. Samples were treated in an ultrasonic bath to remove the grinding material from the surface. No Al_2O_3 was detected during the subsequent microprobe investigation. The samples were investigated promptly after experiments using a Renishaw micro-Raman spectrometer (RM-1000), which is equipped with a Leica DMLM microscope, a 1800 groove per mm grating and a CCD detector. The spectra were obtained using the 532nm emission line of a Nd:YAG laser or the 633nm emission line of a HeNe laser that were calibrated using the 519 cm^{-1} band of a Si wafer. Spectra were collected over several accumulations operating at 10-50% laser power with 3-6min acquisition time for each scan. Analysis of fluid inclusions was mostly performed in confocal mode, while other measurements were made in non-confocal mode to investigate the olivine host for the inclusions. Large areas of each sample were investigated and in several cases additional measurements were made after repeated regrinding and polishing, allowing the sample to be sequentially investigated in the third dimension.

The run products, including the Ir-Fe redox sensors were chemically analysed using a JEOL JXA-8900 Superprobe at the University Frankfurt am Main. Analyses were performed at 15kV and 20nA for the silicate phases and at 20kV and 20nA for the Ir-Fe alloys employing a 1-3µm diameter beam. Fayalite, forsterite, wollastonite, albite, $KTiPO_4$ as well as pure Ir, Fe, Au and Ni were used as standards and peak calibration.

The $Fe^{3+}/\sum Fe$ content of the orthopyroxene starting material was determined by ^{57}Fe Mössbauer spectroscopy on optically clean, hand-picked separates following the procedure given by Woodland et al. [59].

Unpolarised FTIR spectra were obtained on selected samples using a Bruker Tensor 27 infrared spectrometer at the Australian National University in Canberra. Samples were prepared as doubly-polished 80-200 µm thick sections of the capsule assemblage to avoid the presence of fluid inclusions, During analysis, the sample chamber was purged with dry air to minimise interferences, while the background information was collected prior to each analysis. The crystal thickness was calculated by integrating the absorbance in the silicate overtone between 1625 and 2150 cm^{-1} divided by the 0.553 coefficient for unpolarised measurements [60].

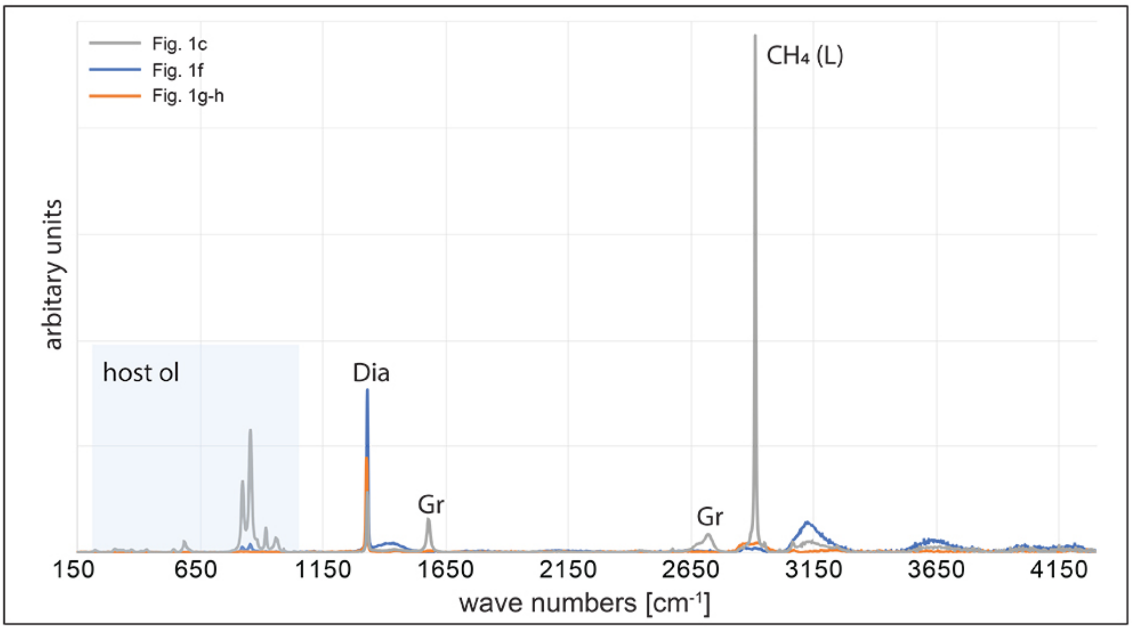

Figure S3: Raman spectra documenting the occurrence of diamonds depicted in Figures 1c,f g-h. Note that diamond is not in contact with graphite. Diamonds in figures f-h are exposed to the atmosphere. Therefore, the absence of CH_4 peak and/or additional presence of H_2O or other higher hydrocarbons can be expected. Unlike fluid inclusion in (c) trapped in olivine, which demonstrates the absence of H_2O and a sharp peak of CH_4.

Other experimental techniques

A number of previous studies with similar starting materials were apparently unsuccessful in producing diamond, such as we observe in our experiments [25, 61, 62]. Although we have no exact answer for these differences, there are number of possible reasons that can be considered. An overriding aspect is the formation and maintenance of CH_4-rich fluids over at least 2 hours [25]. The following factors can influence the fluids stability and composition. (i) Catalytic reaction of fluid with metal capsules (e.g. Fe, Ni, Pd, Pt), as observed and described by Sokol et al.[25], Matveev et al.[63] Matjuschkin et al. [33] leading to fluid disequilibrium and methane instability [33]. In addition, the formation of carbides with Fe and Ni capsules can lead to a net loss of carbon from the fluid (sample). (ii) Hydrogen loss from the sample related to the choice of pressure medium material surrounding the capsule [33,64,65,66,67]. Such loss causes fluid oxidation and disequilibrium. (iii) Configuration of the experimental assembly is important for achieving as close to equilibrium conditions as possible and maintaining the system during the experiment. For instance, the position of H_2-metal buffer is crucial for minimizing the H_2-loss (see above). The inner buffer capsule should not be in contact with external outer capsule in order to prevent direct H_2-diffusion out of the sample (rather than into the sample) [33,67]. On the other hand, placing the buffer external to the sample, can lead to hydrogen-loss from the assembly rather than imparting the fH_2 on the sample [43,62]. (iv) Instable buffering due to use of talc, to produce higher fH_2 [43,61,67]. Dehydration of talk does not guarantee a stable fH_2 in experiment, which can affect the fluid composition. (v) Finally, but not least, an

adequate experimental duration is required in order for solid organic materials (e.g. stearic acid) to produce the fluid phase. Run durations of at least 2 hours appear to be necessary [25,62].

All these aspects together may help to explain the differences between our study and previous work. However, there are maybe additional reasons, which we did not consider here. For example, even the production of the identical assembly may lead to differences due to use of materials from different suppliers (e.g. dense polycrystalline CaF_2 vs. pressed CaF_2 powder [33,67].

Table S1: Starting mix composition obtained by mixing of 40% F7-olivine and 60% F7-orthopyroxene from the Finsch mine, South Africa [36]. Rare Earth Elements (REE) Zr, Hf, Sc, V, Ce,Yb were added to the starting mix in 130 ppm concentrations in form of oxides. b.d.l. = below detection limit.

	SiO_2	TiO_2	Al_2O_3	FeO	Fe_2O_3	MnO	MgO	CaO	Na_2O	K_2O	P_2O_5	NiO	Cr_2O_3	REE
Bulk	51.31	0.01	0.35	4.95	0.23	0.10	42.29	0.29	0.03	b.d.l.	<0.01	0.16	0.19	0.11
Ol	41.51	b.d.l.	b.d.l.	6.71	-	0.09	51.25	0.03	b.d.l.	b.d.l.	b.d.l.	0.41	b.d.l.	-
Opx	57.84	0.02	0.59	3.78	0.39	0.11	36.32	0.47	0.05	b.d.l.	b.d.l.	b.d.l.	0.32	-

Table S2: Summary of experimental conditions and run product compositions where diamond crystallized. Values of $\log fO_2$ were calculated from Ir-Fe redox sensors [34]. Note that additional experiments at 5 GPa that did not produce diamond are reported in Matjuschkin et al. [33]. In all experiments a Mo-MoO_2-H_2O buffer was employed.

exp.	P GPa / T °C	duration, h	Mg# ol/opx	X_{Fe} in Ir-Fe alloy	redox sensor $\log fO_2$	redox sensor ΔIW	detected fluid components
No diamond in experiment							
1583[a]	5/1200	20	93/94	0.35(14)	-9.5(9)	+0.5(9)	CH_4-H_2, HC[b]
1585	5/1250	23	93/94	0.3	-8.66	+0.78	CH_4-H_2, HC[b]
1611	5/1280	12	94/94	0.33	-8.45	+0.47	CH_4-H_2, HC[b]
Diamond bearing experiments (1332cm^{-1} Raman peak confirmed)							
1612	5/1250	15	91/93	0.35	-8.61	+0.5	CH_4-H_2, HC[b]
1632	6/1100	13.5	-	-	-	-	CH_4-H_2, HC[b]
1837	6/1200	11	93/94	0.35	-8.95	+0.64	CH_4-H_2, HC[b]
1633	6/1300	19	-	-	-	-	CH_4-H_2, HC[b]
1805	7/1200	7.5	-	-	-	-	CH_4-H_2, HC[b]
1836	7/1220	7	92/95	0.42	-7.49	+0.23	CH_4-H_2, HC[b]
1838	7/1220	4	94/94	-	-	-	CH_4-H_2, HC[b]
1839	7/1250 to 850	6h run; 2h cooling	92/94	0.37	-	+0.52 to +0.69[c]	CH_4-H_2O-H_2, HC[b]
1766	7/1300	15	93/94	0.35	-7.42	+0.63	CH_4-H_2, HC[b]

2008	7/1300	13	94/94	0.32	-7.78	+0.40	CH_4-H_2, HC[b]
2007	7/1300	10	93/94	0.32	-7.66	+0.52	CH_4-H_2, HC[b]

Conservative uncertainty of fO_2 measurements is ±0.05 log units, as determined by the standard deviation calculated from 5 to 20 analyses in each sample.

[a] Fe-FeO buffered experiment [33]

[b] Unspecified higher hydrocarbons, including C_2H_6

[c] Estimated for the temperature range 1250 – 850°C

References:

1. Tappert, R. *et al.*, Subducting oceanic crust: The source of deep diamonds. *Geology* **33**, 565-568 (2005).
2. Kennedy, C.S., Kennedy, G.C., Equilibrium boundary between graphite and diamond. *J. Geophys. Res.* **81**, 2467-2470 (1976).
3. Irifune, T., Kurio, A., Sakamoto, S., Inoue, T., Sumiya, H., Materials - Ultrahard polycrystalline diamond from graphite. *Nature* **421**, 599-600 (2003).
4. Day, H.W. A revised diamond-graphite transition curve. *Am. Mineral.* **97**, 52-62 (2012).
5. Smart, K.A. *et al.*, Diamond growth from oxidized carbon sources beneath the Northern Slave Craton, Canada: A delta C-13-N study of eclogite-hosted diamonds from the Jericho kimberlite. *Geochim. Cosmochim. Ac.* **75**, 6027-6047 (2011).
6. Stachel, T., Luth, R.W. Diamond formation - Where, when and how? *Lithos* **220**, 200-220 (2015).
7. Pal'yanov, Y. N., Sokol, A.G., Borzdov, Y.M., Khokhryakov, A. F., Sobolev N. V. Diamond formation from mantle carbonate fluids. *Nature* **400**, 417-418 (1999).
8. Stachel, T., Harris, J. W. Diamond precipitation and mantle metasomatism - evidence from the trace element chemistry of silicate inclusions in diamonds from Akwatia, Ghana. *Contrib. Mineral. Petrol.* **129**, 143-154 (1997).
9. Cartigny, P., Palot, M., Thomassot, E., Harris, J. W. Diamond Formation: A Stable Isotope Perspective. *Annu. Rev. Earth Pl. Sc.*, **42**, 699-732 (2014).
10. Thomassot, E., Cartigny, P., Harris, J. W., Viljoen, K. S. F. Methane-related diamond crystallization in the Earth's mantle: Stable isotope evidences from a single diamond-bearing xenolith. *Earth. Planet. Sc. Lett.* **257**, 362-371 (2007).
11. Deines, P. The carbon isotopic composition of diamonds - relationship to diamond shape, color, occurence and vapor composition. *Geochim. Cosmochim. Ac*. **44**, 943-961 (1980).
12. Luth, R.W. Carbon and carbonates in the mantle. *Geo. Soc. S. P*.**6**, 297-316 (1999)
13. Stachel, T., Harris, J. W. Formation of diamond in the Earth's mantle. *J. Phys-Condens. Mat.* **21**, 364206 (2009).
14. Palyanov, Y., Sokol, A.G. The effect of composition of mantle fluids/melts in diamond formation processes. *Lithos* **112,** 690-700 (2009)
15. Frost, D.J., McCammon, C.A. The redox state of Earth's mantle. *Annu. Rev. Earth Planet. Sci.* **36**, 389-420 (2008).
16. Eggler, D.H., Backer, D.R. Reduced volatiles in the system C-O-H: implications to mantle melting, fluid formation, and diamond genesis. *High-pressure research in geophysics*, 237-250 (1982)
17. Leost, I., Stachel, T., Brey, G.P., Harris, J.W., Ryabchikov, I.D., Diamond formation and source carbonation: mineral associations in diamonds from Namibia. *Contrib. Mineral. Petrol.* **145**, 15-24 (2003).
18. Brenker, F.E. *et al.*, Carbonates from the lower part of transition zone or even the lower mantle. *Earth Planet. Sc. Lett.* **260**, 1-9 (2007).
19. Stagno, V., Ojwang, D.O., McCammon, C.A., Frost, D.J. The oxidation state of the mantle and the extraction of carbon from Earth's interior. *Nature* **493**, 84 (2013).
20. Girnis, A.V., Brey, G.P., Bulatov, V.K., Hofer, H.E., Woodland, A.B., Graphite to diamond transformation during sediment-peridotite interaction at 7.5 and 10.5 GPa. *Lithos* **310**, 302-313 (2018).
21. Smart, K.A., Cartigny, P., Tappe, S., O'Brien, H., Klemme, S., Lithospheric diamond formation as a consequence of methane-rich volatile flooding: An example from

diamondiferous eclogite xenoliths of the Karelian craton (Finland). *Geochim. Cosmochim. Ac.* **206**, 312-342 (2017).
22. Smith, E.M. *et al*., Blue boron-bearing diamonds from Earth's lower mantle. *Nature* **560**, 84 (2018).
23. Smit, K.V., Shirey, S.B., Stern, R.A., Steele, A., Wang, W.Y. Diamond growth from C-H-N-O recycled fluids in the lithosphere: Evidence from CH_4 micro-inclusions and delta C-13-delta N-15-N content in Marange mixed-habit diamonds. *Lithos* **265**, 68-81 (2016).
24. Smith, E.M. *et al*., Large gem diamonds from metallic liquid in Earth's deep mantle. *Science* **354**, 1403-1405 (2016).
25. Sokol, A.G. *et al*., Carbon and Nitrogen Speciation in N-poor C-O-H-N fluids at 6.3 GPa and 1100-1400°C, *Sci. Rep.* **7,** 1-19 (2017)
26. Ardia, P., Hirschmann, M.M., Withers, A.C., Stanley, B.D. Solubility of CH_4 in a synthetic basaltic melt, with applications to atmosphere-magma ocean-core partitioning of volatiles and to the evolution of the Martian atmosphere. *Geochim. Cosmochim. Ac.* **114**, 52-71 (2013).
27. Foley, S.F. A Reappraisal of Redox Melting in the Earth's Mantle as a Function of Tectonic Setting and Time. *J. Petrol.* **52**, 1363-1391 (2011).
28. Zhang, C., Duan, Z.H. GFluid: An Excel spreadsheet for investigating C-O-H fluid composition under high temperatures and pressures. *Comput. Geosci.* **36**, 569-572 (2010).
29. Jaques, A. L., et al. "Composition of crystalline inclusions and C-isotopic composition of Argyle and Ellendale diamonds." *International Kimberlite Conference: Extended Abstracts*. Vol. 4. 1986.
30. Rohrbach, A., Ghosh, S., Schmidt, M.W., Wijbrans, C.H., Klemme, S. The stability of Fe-Ni carbides in the Earth's mantle: Evidence for a low Fe-Ni-C melt fraction in the deep mantle. *Earth. Planet. Sc. Lett.* **388**, 211-221 (2014).
31. Akaishi, M., Kumar, M. D. S., Kanda, H., Yamaoka, S., Reactions between carbon and a reduced C-O-H fluid under diamond-stable HP-HT condition. *Diam. Relat. Mater.* **10**, 2125-2130 (2001).
32. Sokol, A.G., Palyanova, G.A., Palyanov, Y.N., Tomilenko, A.A., Melenevsky, V.N. Fluid regime and diamond formation in the reduced mantle: Experimental constraints. *Geochim. Cosmochim. Ac.* **73**, 5820-5834 (2009).
33. Matjuschkin, V., Woodland, A.B., Yaxley, G.M., Methane-bearing fluids in the upper mantle: an experimental approach. *Contrib. Mineral. Petrol.* **174**, (2019).
34. Woodland, A.B., O'Neill, H.S. Thermodynamic data for Fe-bearing phases obtained using noble metal alloys as redox sensors. *Geochim. Cosmochim. Ac.* **61**, 4359-4366 (1997).
35. Yaxley, G.M., Berry, A.J., Kamenetsky, V.S., Woodland, A.B., Golovin, A.V. An oxygen fugacity profile through the Siberian Craton - Fe K-edge XANES determinations of Fe3+/∑ Fe in garnets in peridotite xenoliths from the Udachnaya East kimberlite. *Lithos* **140**, 142-151 (2012).
36. Lazarov, M., Woodland, A.B., Brey, G.P. Thermal state and redox conditions of the Kaapvaal mantle: A study of xenoliths from the Finsch mine, South Africa. *Lithos* **112**, 913-923 (2009).
37. Belonoshko, A.B., Saxena,S.K. A unified equation of state for fluids of C-H-O-N-S-Ar composition and their mixtures up to very high temperatures and pressures. *Geochim. Cosmochim. Ac.* **56**, 3611-3626 (1992).
38. Kerrick, D.M., Jacobs, G.K. A modified Redlich-Kwong equation for H_2O, CO_2, and H_2O-CO_2 mixtures at elevated pressures and temperature. *Am. J. Sc.* **281**, 735-767 (1981).
39. Huizenga, J.M. Thermodynamic modelling of C-O-H fluids. *Lithos* **55**, 101-114 (2001).

40. Lamadrid, H.M., Lamb, W.M., Santosh, M., Bodnar, R.J. Raman spectroscopic characterization of H2O in CO_2-rich fluid inclusions in granulite facies metamorphic rocks. *Gondwana Res.* **26**, 301-310 (2014).
41. Tollan, P.M.W., Smith, R., O'Neill, H.S.C., Hermann, J. The responses of the four main substitution mechanisms of H in olivine to H_2O activity at 1050 degrees C and 3 GPa. *Prog. Earth Planet. Sc.* **4**, (2017).
42. Smith, D.C., Godard, G. A Raman spectroscopic study of diamond and disordered sp3-carbon in the coesite-bearing Straumen Eclogite Pod, Norway. *J. Metamorph. Geol.* **31**, 19-33 (2013).
43. Litasov, K.D., Shatskiy, A., Ohtani, E. Melting and subsolidus phase relations in peridotite and eclogite systems with reduced C-O-H fluid at 3-16 GPa. *Earth Plan. Sc. Lett.* **391**, 87-99 (2014).
44. Tollan, P., Hermann, J. Arc magmas oxidized by water dissociation and hydrogen incorporation in orthopyroxene. *Nat. Geosci.* **12**, 667 (2019).
45. Tollan, P.M.E., O'Neill, H.S. Hermann, J. The role of trace elements in controlling H incorporation in San Carlos olivine. *Contrib. Mineral. Petrol.* **173**, (2018).
46. Peslier, A.H., Woodland, A.B., Bell, D.R., Lazarov, M. Olivine water contents in the continental lithosphere and the longevity of cratons. *Nature* **467**, 78-U108 (2010).
47. Yarbrough, W.A., Messier, R. Current issues and problems in the chemical vapor-deposition of diamond. *Science* **247**, 688-696 (1990).
48. Lee, S.T., Apai, G. Surface phonons and CH vibrational-modes of diamond (100) and (111) surfaces. *Phys. Rev. B* **48**, 2684-2693 (1993).
49. Martinez, J.I., Martin-Gago, J.A., Cernicharo, J., de Andres, P.L. Etching of Graphene in a Hydrogen-rich Atmosphere toward the Formation of Hydrocarbons in Circumstellar Clouds. *J. Phys. Chem. C* **118**, 26882-26886 (2014).
50. Waqar, Z. Hydrogen accumulation in graphite and etching of graphite on hydrogen desorption. *J. Mater. Sci.* **42**, 1169-1176 (2007).
51. Galvez, M.E. *et al.* Graphite formation by carbonate reduction during subduction. *Nature Geosci.* **6**, 473-477 (2013).
52. Brovarone, A.V. *et al.* Massive production of abiotic methane during subduction evidenced in metamorphosed ophicarbonates from the Italian Alps. *Nat. Comm*. **8** (2017).
53. Piccoli. F. *et al.* Subducting serpentinites release reduced, not oxidized, aqueous fluids. *Sci. Rep.* **9**, 19573 (2019)
54. Shirey, S.B. *et al.* Diamonds and the Geology of Mantle Carbon. *Rev.Mineral. Geochem.* **75**, 355-421 (2013).
55. Yang, X., Keppler, H., Li, Y. Molecular hydrogen in mantle minerals. *Geochem. Perspect.* **2**, 160 (2016).
56. Berry, A.J., O'Neill, H.S.C., Hermann, J., Scott, D.R. The infrared signature of water associated with trivalent cations in olivine. *Earth Planet. Sc. Lett.* **261**, 134-142 (2007).
57. Sokol, A.G., *et al.* Formation of water bearing defects in olivine in the presence of water-hydrocarbon fluid at 6.3 GPa and 1200°C. *Dokl. Earth Sci.* **483** (2018)
58. Akella, J., Kennedy, G. C. Melting of gold, silver and Copper - proposal for a new high-pressure calibration scale. *J. Geophys. Res.* **76**, 4969-& (1971).
59. Woodland, A.B., Kornprobst, J., Tabit, A. Ferric iron in orogenic lherzolite massifs and controls of oxygen fugacity in the upper mantle. *Lithos* **89**, 222-241 (2006).
60. Shen, T.T., Hermann, J., Zhang, L.F., Padron-Navarta, J.A., Chen, J. FTIR spectroscopy of Ti-chondrodite, Ti-clinohumite, and olivine in deeply subducted serpentinites and implications for the deep water cycle. *Contrib. Mineral. Petrol.* **167** (2014).

61. Sokol, A.G. *et al.* Fluid regime and diamond formation in the reduced mantle: experimental constrains. *Geochim. Cosmochim. Ac.* **73** (2009)
62. Sokol, A.G. *et al.* Hydrogenation of carbon at 5.5-7.8 GPa and 1100-1400°C: implications to formation of hydrocarbons in reduced mantles of terrestrial planets. *Phys. Earth Planet. In.* **291** (2019)
63. Matveev, S. *et al.* Volatiles in the Earth's mantle. I. Synthesis of CHO fluids at 1273 K and 2.4 GPa. *Geochim. Cosmochim. Ac.* **61** (1997)
64. Eugster, H.P. Heterogeneous reactions involving oxidation and reduction at high pressures and temperature. *J. Chem. Phys.* **26** (1957)
65. Truckenbrodt, J., Ziegenbein, D., Johannes, W. Redox conditions in piston-cylinder apparatus: the different behaviour of boron nitride and unfired pyrophyllite assemblies. *Am. Mineral.* **82** (1997)
66. Jakobsson, S. Oxygen fugacity control in piston cylinder experiments, *Contrib. Mineral. Petrol.* **164** (2012)
67. Matjuschkin, V. *et al.* Control and monitoring of oxygen fugacity in piston cylinder experiments. *Contrib. Mineral. Petrol.* **169**, (2015)
